# Adenylosuccinic Acid: An Orphan Drug with Untapped Potential

**DOI:** 10.3390/ph16060822

**Published:** 2023-05-31

**Authors:** Emma Rybalka, Stephanie Kourakis, Charles A. Bonsett, Behzad Moghadaszadeh, Alan H. Beggs, Cara A. Timpani

**Affiliations:** 1 Institute for Health and Sport (IHeS), Victoria University, Melbourne, VIC 8001, Australia; stephanie.kourakis@live.vu.edu.au (S.K.); cara.timpani@vu.edu.au (C.A.T.); 2 Inherited and Acquired Myopathy Program, Australian Institute for Musculoskeletal Science (AIMSS), St Albans, VIC 3021, Australia; 3 Department of Medicine—Western Health, Melbourne Medical School, The University of Melbourne, St Albans, VIC 3021, Australia; 4 Division of Neuropaediatrics and Developmental Medicine, University Children’s Hospital of Basel (UKBB), 4056 Basel, Switzerland; 5 Dystrophy Concepts Incorporated, Indianapolis, IN 46226, USA; tcbonsett@att.net; 6 School of Medicine, Indiana University, Indianapolis, IN 46202, USA; 7 The Manton Center for Orphan Disease Research, Division of Genetics and Genomics, Boston Children’s Hospital, Harvard Medical School, Boston, MA 02115, USA; behzad@enders.tch.harvard.edu (B.M.); beggs@enders.tch.harvard.edu (A.H.B.)

**Keywords:** adenylosuccinic acid, adenylosuccinate, succinyl-AMP, Duchenne muscular dystrophy, ADSSL1 myopathy, metabolic disease, skeletal muscle, purine metabolism, immunometabolism, Nrf2 activation

## Abstract

Adenylosuccinic acid (ASA) is an orphan drug that was once investigated for clinical application in Duchenne muscular dystrophy (DMD). Endogenous ASA participates in purine recycling and energy homeostasis but might also be crucial for averting inflammation and other forms of cellular stress during intense energy demand and maintaining tissue biomass and glucose disposal. This article documents the known biological functions of ASA and explores its potential application for the treatment of neuromuscular and other chronic diseases.

## 1. Introduction

In 1984, adenylosuccinic acid (ASA), a purine nucleotide cycle (PNC) intermediate, was used for the first time as an experimental pharmaceutical to combat the fatal neuromuscular disease, Duchenne muscular dystrophy (DMD), and the less severe Becker (BMD) variant [1]. Phase I safety and subsequent phase II efficacy and dosage trials were conducted throughout the 1980s by Dr. Charles A. Bonsett MD, Ann Rudman and Dr. Vimal Patel PhD from the Department of Neurology, Indiana University School of Medicine. Bonsett’s team had spent the decades prior characterizing DMD from an anatomical and biochemical perspective before the inherited *DMD* gene (encoding dystrophin) and its mutations were discovered. They described several nuances of DMD muscles indicating metabolic disease, including the empirical observations that: (1) DMD myofibers were tacky due to surface lipid accumulation, which was traced back to the focal dysfunction of the tricarboxylic acid (TCA) cycle enzyme, isocitrate dehydrogenase (IDH), a significant control point of mitochondrial adenosine triphosphate (ATP) synthesis [2,3]; and that this liposis was associated with (2) the loss of muscle elastic elements [4] and (3) fibrosis of the extracellular matrix (ECM) [4]. It was hypothesized that supplemental metabolites could be of medicinal value to alleviate these symptoms.

ASA was selected for clinical testing based on the positive in vitro screening of several different metabolites. A trace addition of ASA, but not other purine nucleotides (e.g., adenosine monophosphate (AMP), adenosine diphosphate (ADP), ATP, inosine monophosphate (IMP)), to cultured human DMD muscle explants corrected the mitochondrial TCA cycle defect, and could prevent lipid biosynthesis and secretion [2]. An intravenous infusion of DMD patients reduced disease biomarkers (e.g., blood creatine kinase (CK) levels, muscle degeneration-to-regeneration ratio, inflammatory infiltrate, adiposis and fibrosis) proportionate to incremental dosage escalation (30–300 mg/kg.bw/day) [1]. The trials, which ran over more than a decade, were eventually abandoned when the *DMD* gene defect and missing protein product (dystrophin) were discovered [5,6]. DMD was proven to be a disease of membrane fragility due to dystrophin deficiency, rather than an inborn error of metabolism, and funding was directed towards the development of curative gene-targeted therapeutics. ASA, which was expensive to manufacture and difficult to source, became an orphan drug despite evidence of therapeutic value in a disease that has now been characteristically linked to chronic, pathological inflammation and hyper-immunity [7], as well as deregulation of the cytosolic and mitochondrial metabolic systems [8]. Ironically, using modern “big data” omics techniques, adenylosuccinate synthetase (ADSS) expression has recently been shown to be as significantly downregulated in DMD muscle as in the primary ADSS-deficient disease, ADSSL1 myopathy [9].

Although traditionally regarded as mere by-products of metabolism, metabolites are now understood to have important signaling functions, both within cells and in distant tissues and organs, following their release into the extracellular space and/or blood [10]. Since cell life depends on the adenylate charge, metabolism is especially linked to cell fate signaling and onward to immune system function in a concept known as immunometabolism [11]. Metabolites and reactive oxygen species (ROS) play instrumental roles within this immunometabolic nexus and purine nucleotide metabolism appears particularly influential since the loss of the function of the governing enzymes leads to progressive disease often with severe immunological manifestations [12]. For example, the impaired function of the fatty acid metabolism-immunity nexus (FAMIN), an enzyme shown recently to metabolize purine nucleosides using ASA, is associated with severe immunologic disease including juvenile idiopathic arthritis, Still’s disease, early onset inflammatory bowel disease, Crohn’s disease and leprosy [13]. Therefore, the pharmacological manipulation of immunometabolic regulatory checkpoints represents a promising strategy for the management of disease driven by metabolic and/or immunologic deregulation. ASA was recently shown to activate the master cytoprotective transcription factor, nuclear factor 2-erythroid-like factor 2 (Nrf2/NFE2L2), following the moderate-term treatment of mice [14] and to reduce the ROS content in myoblast culture [15]. ASA metabolism generates fumarate as a by-product, which is a well-established activator of the endogenous antioxidant response via Nrf2 induction [16]. Cytosolic fumarate metabolism is also essential for the DNA repair mechanisms that promote cell survival [17]. In this paper, we explore the idea that ASA could be a useful drug capable of both functional metabolic reprogramming, cytoprotection and immunosuppression. As such, it could be applied to a variety of diseases as a useful pharmacological tool.

## 2. ASA: Biological Activity and Pharmacology

**Figure 1:**
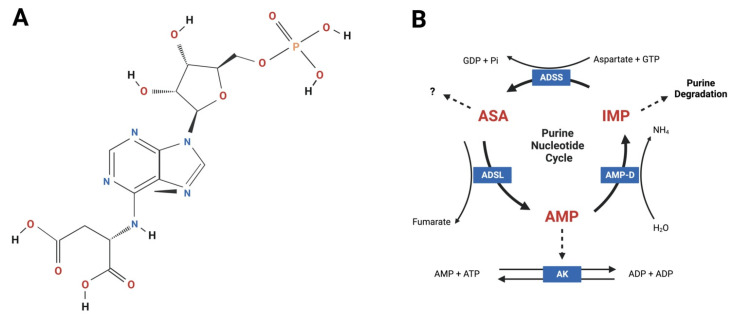
Adenylosuccinic acid (ASA) is an aromatic small molecule that functions within the purine nucleotide cycle (PNC) metabolon. (**A**) Chemical structure of ASA according to [18]; (**B**) ASA is endogenously synthesized by adenylosuccinate synthetase (ADSS) using inosine monophosphate (IMP), aspartate and guanosine triphosphate (GTP). Guanosine diphosphate (GDP) and inorganic phosphate (Pi) are generated as by-products of this reaction. ASA is catalyzed by adenylosuccinate lyase (ADSL) generating adenosine monophosphate (AMP) and fumarate. Under low energy conditions, AMP exits the PNC and enters the adenylate kinase (AK) reaction to generate two molecules of ADP that are transported across the mitochondrial membranes via adenine nucleotide translocase for re-phosphorylation by ATP synthase (Complex V). When cellular energy status is high, AMP is catalyzed to IMP, which is further degraded and eliminated from cells producing ammonia (NH_4_). The PNC facilitates energy balance, mitochondrial coupling via fumarate-aspartate exchange and excretion of nitrogenous waste. Thick solid lines = reactions; thin solid lines = cofactors and products; broken lines = PNC exit pathways. Figure created using Biorender.com.

ASA (synonyms: adenylosuccinate, *N*6–(1,2-dicarboxyethyl)–AMP, aspartyl adenylate, succinyl AMP) is a naturally occurring aromatic heteropolycyclic small molecule belonging to the purine ribonucleoside monophosphate class of organic compounds (chemical formula C_14_H_18_N_5_O_11_P; molecular weight 463.2934 [18,19]; Figure 1A). It is found in all cell types within the PNC metabolon (Figure 1B) but appears especially important in tissues subject to dynamic fluctuations in energy demand, including skeletal and cardiac muscle, brain, kidney and liver [20]. Endogenous ASA is derived from inosinic acid (specifically IMP) and aspartate in a reaction catalyzed by ADSS [21]. It is subsequently converted to AMP and fumarate via the catalytic activity of adenylosuccinate lyase (ADSL) [22]. The principal biological function of ASA metabolism is to (1) balance cellular ATP levels and energy homeostasis during fluctuations in energetic demand; and (2) convert potentially toxic ammonia into inert glutamine for release into the blood stream and excretion by the kidneys [20,23]. To this effect, the PNC represents a simplistic enzyme system geared toward safely salvaging IMP from further degradation, and eventually, elimination from the cell (discussed in detail later; Figure 1B). However, ASA has emerged as a critical metabolic checkpoint with more complex and widespread biological functions than originally thought. Predominantly through its capacity to generate fumarate and sequester aspartate, ASA metabolism synchronizes the rate of purine nucleotide degradation—which is directly reflective of ATP demand—to mitochondrial TCA cycle capacity via the malate–aspartate shuttle. Cytosolic fumarate hydratase (FH) converts fumarate to malate to enact this shuttle and, in doing so, triggers pseudo-hypoxia stress signaling via the stabilization of hypoxia inducible factor 1 (HIF-1α and β) [24]. When the mitochondrial oxidative phosphorylation (OXPHOS) capacity is reached and cytosolic FH saturated, ASA-generated fumarate can accumulate in the cytoplasm and exert numerous signaling functions to communicate tissue stress. These include: (1) the modulation of the innate and adaptive immune systems via multiple mechanisms; (2) the rewiring of metabolic networks in favor of lipid over glucose utilization (presumably to facilitate glycogen replenishment); and (3) the induction of the Nrf2ome responsible for anti-oxidative and -inflammatory cytoprotective mechanisms (reviewed in [24]). ASA may also elicit metabolic modulatory activity independently of fumarate. In human Type 2 diabetic pancreatic islet cultures, ASA infusion (10 μM) can restore glucose-stimulated insulin secretion demonstrating secretagogue control over systemic glucose disposal [25]. Furthermore, ASA treatment stimulates total/phosphocreatine pool anaplerosis in skeletal muscle [1,15]. These functions are all geared toward alleviating intense metabolic stress and facilitating the necessary adaptations to promote cell survival in biological systems under pressure.

As an exogenously administered pharmaceutical, there are few data concerning ASA’s pharmacodynamics. Currently, its absorption kinetics, tissue distribution, protein binding, route of elimination, half-life and clearance are unknown. Predictive ADMET modelling suggests ASA is unlikely to cross the gut barrier based on molecular size and polarity [19], and therefore is probably not bioavailable to target tissues. This modelling, though, is in direct opposition with our own pre-clinical work. In healthy C57BL/10 mouse skeletal muscle, orally administered ASA (~325 mg/kg/day over 8 weeks, Sigma 96% pure formulation delivered in reverse osmosis drinking water; pH 7.2): (1) increased the total creatine pool, and more specifically, phosphocreatine concentration, by 20–25%; (2) reduced AMP kinase (AMPK) phosphorylation (by ~50%) and increased ATP content by ~20%, indicative of improved AMP > ADP > ATP recovery; and (3) increased the activity of mitochondrial TCA cycle enzymes, citrate synthase (CS) and succinate dehydrogenase (SDH), by 25%; [15]. We later showed Nrf2 upregulation in the same samples [14]. Our data demonstrate that ASA is not only bioavailable to target tissues via the oral route, but that it integrates within the PNC metabolon to generate AMP and fumarate that enhance energy stores and induce synergistic mitochondrial anaplerosis and cytoprotection, respectively. 

Bonsett’s ASA clinical trial data further demonstrates its bioavailability in the context of humans [1,26]. Initially, ASA (200 mg/mL ASA tetrasodium salt in sodium chloride formulation) was administered to five DMD patients via daily subcutaneous injection to varied sites including the left and right deltoid area, abdomen and gluteus. The subcutaneous route is useful to encourage a more sustained release of the drug compared to the oral route. ASA dose was increased in daily increments of 1 mg/kg b.w., commencing at 1 mg/kg b.w. on day 3, until the phase 1 maximum dose of 10 mg/kg b.w. was reached on day 19, which was maintained to day 28 of observations. Throughout the phase 1 safety trial there were no adverse effects noted. There were also no objective clinical signs of (functional) improvement aside from a higher urinary creatinine:creatine ratio [26], consistent with our pre-clinical data showing ASA-stimulated intramuscular creatine phosphorylation [15]. The subsequent phase II trial escalated the dose and trialed alternative delivery routes/tools. Since dosage was determined empirically based on an estimated daily ATP requirement per kilogram of body mass, a maximum dosage of 600 mg/kg/day (increased from 300 mg/kg/day maximum previously) was ultimately authorized by the FDA as the patients grew over the trial duration [26]. To facilitate this, delivery methods were upgraded from multiple daily subcutaneous injections to an insulin pump and then finally to implantable intraperitoneal port-a-cath [26]. However, the highest dosage ever reached in the trial was 300 mg/kg/day due to technical complications with peritoneal delivery (fibrotic scarring of the abdominal musculature blocked the port-a-cath). By the end of the trial, delivery via the oral route (5 g twice daily in juice) was used to achieve a higher daily dosage resulting in strength improvements in one patient and maintenance of biofluid disease markers in another [3]. Over the decade long trial, there were no adverse effects associated with chronic ASA treatment and disease progression was significantly slowed as measured by clinical blood biomarker of muscle necrosis, CK, the urinary creatinine:creatine ratio and the distal (finger) muscle strength test. Notably, plasma/serum CK levels are prone to fluctuation in DMD and can markedly drop due to muscle loss in advanced disease [27]. However, compellingly, in one patient with slower, atypical onset (but confirmed) DMD [28], serum CK levels were attenuated by increasing ASA dosage; 73 mg/kg/day ASA saw CK levels of 1000–2000 U/L (normal range 20–200 U/L) whereas, when ASA treatment was periodically discontinued for 6 months, CK levels were elevated to ~20,000 U/L [26].

**Figure 2:**
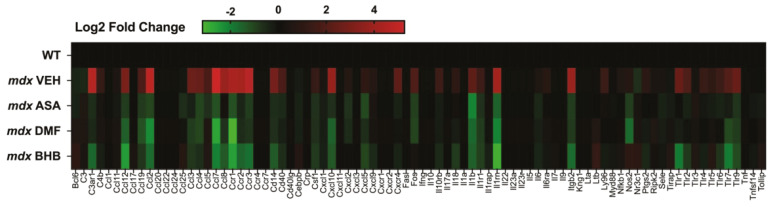
Inflammatory gene signatures of murine Duchenne muscular dystrophy (DMD) skeletal muscle treated with and without hydroxycarboxylic acid receptor 2 (HCAR2) agonist immunomodulatory metabolites. Juvenile (14-day-old) wild-type (WT) and *mdx* mice were treated with either a methyl cellulose (MC) vehicle, or 325 mg/kg/day ASA, 100 mg/kg/day DMF or 380 mg/kg/day BHB suspended in MC via oral gavage for 2 weeks. Quadriceps muscles were harvested under non-recovery anesthesia and the inflammatory gene signature was profiled using qPCR array (for methodology see Supplemental information). Data shown are log2 fold change expression from WT.

The question remains, if ASA is too large and polarized to permeate biological membranes via simple diffusion then how does it cross the gut epithelium and the membranes of target tissues? Indeed, there is no study to date that has investigated ASA flux across membranes to identify the precise transport mechanism. Structurally similar, albeit smaller, metabolites such as fumarate and citrate are capable of crossing the gut interstitial brush border via a class of transporters specific for tri- and dicarboxylates [29]. Dicarboxylic acid transporters have also been identified on vascular smooth muscle membranes and enable the dynamic control of metabolic flux and reprogramming based on available blood substrates [30]. ASA might permeate the gut wall in a similar mechanism or, alternatively, be converted to fumarate within the gut epithelium or via the gut microbiome and circulated in this form. Mammalian metabolite transport is mostly facilitated through members of the solute carrier (SLC) family of membrane transporters. There are many SLC transporters with unknown substrates and function [31] and it is possible that one might be specific for distributing circulating ASA to target tissues and vice versa. Another alternative/synergistic mechanism postulated by us previously is that ASA binds the hydroxycarboxylic acid receptor 2 (HCAR2/GPR109A)—a g-coupled cell surface receptor with diverse functions [14]. The metabolite HCAR2 agonist, β-hydroxybutyrate (BHB), activates SIRT1 to induce many pro-survival adaptations including mitochondrial biogenesis, anti-oxidation, autophagy, metabolic reprogramming, increased ATP production and inhibition of apoptosis (reviewed in [32]). Through HCAR2, BHB also inhibits the master controller of inflammation, nuclear factor kappa B (NFκB), to suppress cytokine production and the immune system [32]. Fumarate esters, such as dimethyl fumarate (DMF) activate HCAR2 as well as Nrf2 once inside the cell. Our data generated in the *mdx* mouse model of DMD demonstrate that oral ASA (325 mg/kg/day), DMF (100 mg/kg/day) and BHB (380 mg/kg/day) induce very similar anti-inflammatory gene profiles (Figure 2), suggesting that at least part of ASA’s mechanism of action is via the agonism of HCAR2.

## 3. Multi-Functions of ASA: A Deeper Perspective

### 3.1. ASA Metabolism Synergises Mitochondrial and Cystosolic Energy Systems

Purines form DNA, RNA and ATP (and lower phosphate analogues), molecules essential for cell maintenance, repair, replication and protein synthesis. Since cell life depends on these building blocks and the adenylate charge, unique enzyme systems have evolved to salvage degrading purines before they flux from cells (i.e., the PNC), and to replenish the purine nucleotide pool through de novo biosynthesis when depletion is inevitable [20]. Both systems involve ADSL, which cleaves fumarate from ASA to be sequestered into the mitochondrial TCA cycle. The most accepted transport mechanism is via the malate–aspartate shuttle, which first requires the conversion of ADSL-generated fumarate to malate via cytosolic FH activity [20]. Fumarate might also be directly transported in mammalian cells by a mitochondrial dicarboxylic acid transporter as it is in yeast [33]. In this manner, ADSL-generated fumarate facilitates a biochemical coupling between the cytosolic and mitochondrial metabolons, synchronizing them to meet dynamic fluctuations in ATP demand. 

**Figure 3:**
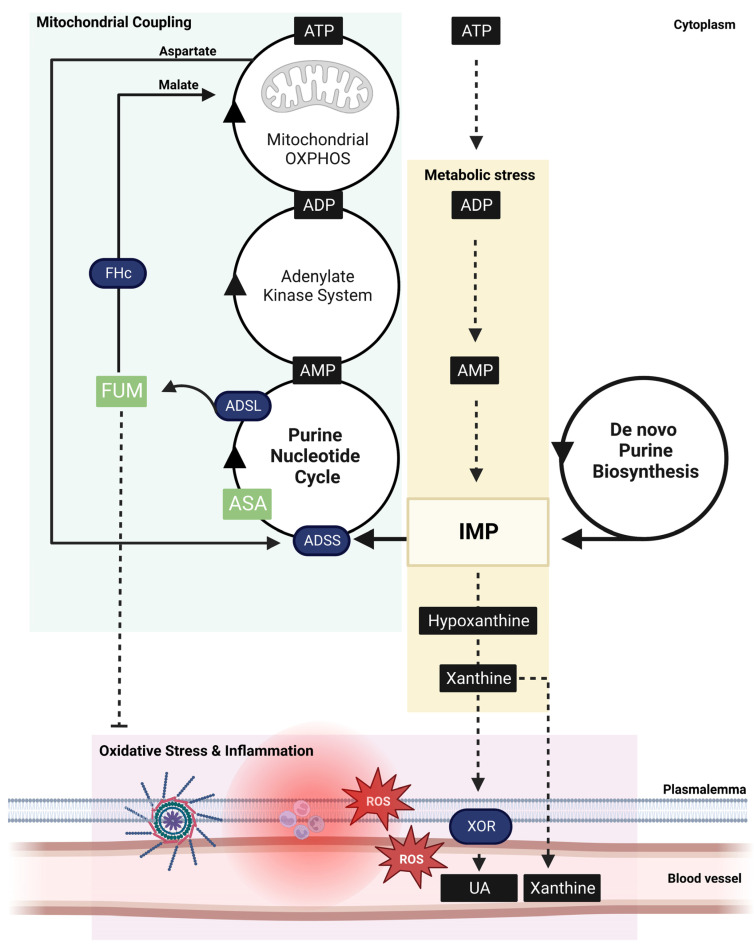
Schema of the critical role adenylosuccinic acid (ASA) plays in coupling cellular metabolic stress signals with mitochondrial function. During intense energy expenditure (exercise, cell damage, tissue repair) or metabolic disease, adenosine triphosphate (ATP) is rapidly degraded to inosine monophosphate (IMP) which, if not sequestered into the purine nucleotide cycle (PNC), leads to further degradation to hypoxanthine and xanthine (yellow box). The PNC sequesters IMP into a reaction with GDP catalyzed by adenylosuccinate synthetase (ADSS) generating ASA. ASA is subsequently catalyzed to AMP and fumarate (FUM), a key step in ensuring cytosolic energy demand is coupled to mitochondrial ATP synthesis via oxidative phosphorylation (OXPHOS; green box). FUM is converted to malate by cytosolic fumarate hydratase (FHc), which is exchanged across the double mitochondrial membrane for aspartate via the malate–aspartate shuttle. Malate drives the mitochondrial tricarboxylic acid cycle, a key generator of NADH that supports OXPHOS-mediated conversion of ADP derived from the linked PNC-adenylate–kinase system. Aspartate fuels ASA synthesis within the PNC at a matched rate. When energy expenditure exceeds the capacity of this coupled system (which can be compromised by metabolic disorders), IMP is diverted away from the PNC and rapidly degraded to hypoxanthine and xanthine, which is extruded from cells via xanthine oxidoreductase (XOR) metabolism as uric acid (UA). XOR is a rampant producer of reactive oxygen species (ROS), which drives oxidative stress and inflammation (pink box). ASA-generated FUM can suppress oxidative stress and inflammation via activation of cytoprotective transcription factor, Nrf2. During critical ATP shortage, purines can be de novo biosynthesized in a slow, and bioenergetically expensive, process yielding IMP. ASA is still required to convert IMP to usable bioenergy (AMP > ADP > ATP) and is therefore staple in both purine salvage and biosynthesis pathways.

De novo purine biosynthesis is dependent upon several supportive metabolons including folate, ribose (derived from glucose metabolism) and polyamine metabolism and, equivocally, is slow and bioenergetically expensive [34]. Thus, it is used as a “last resort” to buffer critically low ATP levels that are incompatible with cell life. Within this enzyme system, ADSL converts 5-aminoimidazole-(N-succinylocarboxamide) ribotide (SAICAR) to 5-aminoimidazole-4-carboxamide ribotide (AICAR) and fumarate. AICAR is subsequently used to generate IMP [34]. The PNC recycling/salvage pathway has two important functions regarding purine homeostasis. Firstly, it recovers degrading purines before they leach from cells, thus alleviating the dependency on de novo biosynthesis. Within this enzyme system ADSL captures IMP before it is further degraded to nitrogen bases, into a catalytic reaction with ASA to produce AMP and fumarate [20]. Secondly, via the same reaction, it processes de novo IMP to complete purine biosynthesis [34]. Whether IMP is de novo synthetized by ADSL from AICAR, or captured by the PNC from further degradation reactions, ADSS activity and ASA are required to convert it to a usable cellular energy form (i.e., AMP; Figure 3) [20]. 

Disorders of purine nucleotide metabolism are problematic, not only because of increased purine degradation and flux from tissues, but because coupling between energy demand and the mitochondrial capacity to meet that demand diminishes. When ADP is progressively degraded to AMP, adenine nucleotide translocator (ANT) function across the mitochondrial membrane is lost and so, too, is the principal stimulus for mitochondrial OXPHOS capacity [35]. Similarly, when AMP is progressively degraded to IMP, the activation of the critical stress response induced by AMPK is diminished—this response typically functions to increase mitochondrial biogenesis, suppress protein synthesis and consequently ATP demand, instigate macro-autophagy to increase substrate availability for energy synthesis, and orchestrate cell stress signaling, especially anti-oxidation and metabolic reprogramming mechanisms (for an excellent review see [36]). ASA is essential for capturing IMP and recoupling mitochondrial function with cytosolic energy demand via driving the malate–aspartate shuttle. Under low NADH:NAD+ conditions, fumarate is rapidly converted to malate by cytosolic FH [20]. Malate is then exchanged across the mitochondrial membranes for aspartate, in a multi-step process involving several additional enzymatic reactions [20] (Figure 3). Aspartate drives ASA synthesis by ADSS to maintain coupling between the systems. FH is essential to this mechanism and has the additional effect of keeping cytosolic fumarate concentrations low enough to drive maximal TCA cycle activity. When fumarate levels build, protein succination of aconitase occurs and drives the conversion of citrate to neutral fats, instead of to isocitrate, to support OXPHOS [37]. Aside from the reverse reactivity of FH, which occurs upon translocation to the nucleus in response to DNA damage and drives malate > fumarate to stimulate DNA repair (reviewed in [17]), ASA is the only cytosolic generator of fumarate. The loss of endogenous ASA synthesis would theoretically reduce fumarate production, FH activity and malate–aspartate shuttle-mediated PNC-mitochondrial coupling. Pharmacological ASA replenishment could be a useful strategy to re-establish these functions in diseases in which normal ADSS function, and PNC-mitochondrial coupling, is diminished.

### 3.2. ASA Metabolism Suppresses ROS Production and Inflammation

The loss of cellular ATP into the extracellular compartment, which typically occurs during plasmalemma damage, poses an immediate bioenergetical crisis to the cell that is incompatible with survival. Consequently, an intense immune response is triggered through the agonism of purinergic receptors on the outer membrane surface [38]. In this manner, higher order purines act as damage-associated molecular patterns (DAMPs) within the extracellular environment to drive inflammation [39]. Ultimately, this inflammatory response functions to recruit immune cells to the injury site to digest, and then repair, damaged tissue [38]. In a similar manner, the flux of nitrogen-bases (e.g., hypoxanthine (Hpx) and xanthine)—generated as end-products of purine degradation—from cells into the extracellular compartment signals intense metabolic stress and a similar immunological response. When ATP demand exceeds the cells capacity to recycle purines through the salvage pathway, IMP is ultimately degraded, i.e., IMP > inosine > Hpx > xanthine > uric acid (UA) [20], and leached into the extracellular compartment and/or blood stream (Figure 3). The degradation of Hpx > xanthine and xanthine > UA is controlled by xanthine oxidoreductase (XOR), an enzyme mostly expressed in vascular endothelium, but which also circulates in the plasma demonstrating systemic signaling function [40]. The activity of both the dehydrogenase (XDH) and oxidase (XO) isoforms of XOR results in ROS production. XO reduces O_2_ bivalently or univalently to produce hydrogen peroxide (H_2_O_2_) or superoxide anion (O_2_^−^), respectively [41]. In contrast, XDH produces ROS during the oxidation of NADH at the FAD-containing domain depending on the pH and O_2_ tension of the cell [41]. XO activity corresponds with increased vascular permeability and NRLP3 inflammasome assembly and activity [42,43]. The magnitude and duration of this inflammatory response is mostly dependent upon the XO isoform predominance, which is controlled by calcium-dependent proteolysis, the oxidation of cysteine residues and/or the NAD+:NADH ratio [44]. All of these are biochemical indicators of metabolic stress.

In physiological ranges, ROS produced by XO are signaling molecules responsible for eliciting hormetic adaptations, mostly through the activation of the Nrf2-mediated cytoprotective program [44]. Nrf2 induces the phase II anti-oxidative response resulting in an increased expression of ROS-neutralizing enzymes and the inhibition of NF_Κ_B-mediated inflammation. However, in pathological inflammatory states, XO activity drives several mechanisms that result in tissue injury, inflammation and chronic immune system activation, e.g., gout, rheumatoid arthritis (reviewed in [40]). UA, the product of xanthine degradation, has anti-oxidative properties which can buffer XO-generated ROS [45]. This mechanism also assists in delaying immunologic activation. However, when XO enzymes become saturated or inhibited pharmacologically by allopurinol, HPx can: (1) still be degraded to xanthine; and (2) flux from cells into blood and be excreted through the renal system without conversion to UA. In this situation, ROS are still produced through the first XO reaction, but at a rate that exceeds the antioxidant capacity of UA (produced in the second XO reaction) resulting in oxidative stress [40]. This oxidative stress likely acts as a tipping point in the immunometabolic nexus. During systemic inflammation, the redox status of UA may already be challenged through the intensity of ROS-production by immune cells, thus increasing the rate of unbuffered ROS production driven by purine degradation to perpetuate inflammation in a feed-forward mechanism. 

In pathological states where endogenous ASA production is compromised, e.g., in metabolic/mitochondrial disease (especially ADSS-deficiency), and chronic and severe metabolic stress are induced, there are multiple opportunities for oxidative stress and chronic immune system activation to perpetuate. In the first instance, the increased production of XO substrates, xanthine and HPx, would escalate ROS production [44]. Secondly, a reduced cytosolic fumarate production may dampen Nrf2 induction and the cellular armor to buffer XO-mediated ROS production. Thirdly, the stress-adaptive effects induced by FH activity, e.g., angiogenesis and metabolic reprogramming, would become repressed due to the reduced fumarate production. DMD provides a good context for this theory. DMD patients have elevated skeletal muscle XO activity compared to age-matched controls, which corresponds with the increased urinary oxidative stress biomarker, ortho-tyrosine [46]. XO is also hyperactive in skeletal muscles of *mdx* mice carrying the same genetic defect as human DMD patients, resulting in increased oxidative stress biomarkers in blood [46]. This XO hyperactivity corresponds with a loss of contraction force [47], suggesting that the inhibitory role of XO-generated O_2_^−^ on muscle function might be protective in order to limit the energy demand in a system under intense metabolic pressure. By limiting energy expenditure, and thereby further purine loss, inflammation can be curtailed, and energy re-directed to muscle re-modelling/repair processes. As DMD progresses, Nrf2 activation is also significantly dampened despite escalating ROS production and oxidative stress [48], suggesting that ASA-generated fumarate production might be essential for the appropriate dose-response induction of the Nrf2ome.

There is tremendous potential for ASA to be therapeutically useful as an alternative to XOR blockade by drugs such as allopurinol, febuxostat and topiroxostat, and for the treatment of the many diseases associated with XOR hyperactivity. These include gout, autoimmune rheumatic diseases, such as systemic lupus erythematosus and rheumatoid arthritis [49], pre-eclampsia [50], multiple sclerosis [51], cardiovascular disease [52] and metabolic syndrome [53]. Whereas XOR inhibition prevents the HPx > xanthine flux of purines from metabolically stressed tissues, and the associated ROS (and inflammation) production, ASA would protect against the production of these by-products in the first place and alleviate the causal metabolic stress. 

### 3.3. ASA Functions within the Nrf2ome

As introduced earlier, there is both evidence of, and a strong biochemical rationale for, Nrf2 activation by ASA [14]. Keap1 represses Nrf2 at a stochiometric ratio of 2:1 (reviewed in [54]). An equivalent number of fumarate molecules are required to fully dissociate Nrf2 [55] and enable the nuclear translocation and transcription of the antioxidant response element on target genes. ADSL generates two fumarate molecules for every de novo biosynthesis–PNC coupled reaction [20], although that ratio likely fluctuates based on: (1) the dynamic oscillations in energy demand where PNC predominates fundamentally due to the speed of its reactions; (2) the degree to which anaplerosis due to enzyme upregulation predominates in one system over the other—there is currently little understanding of the factors that up-/downregulate purine homeostasis enzymes; and (3) the degree to which anaplerosis, due to substrate availability, predominates in one system over the other—during metabolic stress, purine biosynthesis is inhibited, at least initially, by the mitochondrial consumption of building blocks, whereas substrates for PNC-mediated salvage increase. The loss of mitochondrial coupling when the PNC is maximally activated leads to a rapid build-up of aspartate and fumarate within the cytosol and Nrf2 activation. Simultaneously, ROS produced by XO activity (as well as pressure on the NADH:NAD+ ratio) would co-activate Nrf2. 

Intriguingly, Nrf2 signaling has emerged as an important regulatory switch governing purine levels. Traditionally regarded as a transcription factor that monitors oxidative stress, there is increasing evidence that Nrf2 also senses metabolic stress and orchestrates a response to maintain purine levels. Nrf2 is strongly activated by both ROS (e.g., generated by XO), and fumarate (e.g., generated by the PNC), demonstrating the first-line detection of increased purine degradation and recovery mechanisms via its traditional activators. Nrf2 activity can be artificially stimulated by both (dimethyl/monomethyl/diroximel) fumarate (reviewed in [16]) and ASA (ASA > fumarate) [14] treatment resulting in the modulation of >200 genes associated, not only with anti-oxidation and -inflammation, but also with carbohydrate, lipid and purine metabolism and protein degradation (especially via autophagy) [16]. However, a recent study demonstrated that the therapeutic effect of Nrf2 activity on the progression of murine radiation-induced lung fibrosis was controlled through the piwi-like RNA-mediated gene silencing 2 (*PIWIL2*)-mediated reprogramming of purine metabolism. PIWIL2 controls stem cell proliferation via STAT3 and the epigenetic regulation of metabolism by maintaining histone deacetylase 3 (HDAC3) phosphorylation [56]. Inhibiting key purine biosynthesis enzymes, IMP dehydrogenase 1 and 2 (IMPD1 and 2), abolished the protective effect of Nrf2 signaling on lung fibrosis, demonstrating evidence of a Nrf2/PIWIL2/purine metabolism axis [56]. Nrf2 indirectly modulates several enzymes involved in de novo purine synthesis and directs glucose metabolism toward purine nucleotide synthesis over mitochondrial OXPHOS in proliferating fibroblasts (reviewed in [57]). Nrf2 binds the promoter region on the ADSSL1 gene [58], suggesting that it may directly control ASA production capacity and, consequently, fumarate synthesis, through upregulating the transcription of ADSS in a feed-forward mechanism. This idea needs to be confirmed. 

As reviewed by us previously, Nrf2 is an exciting drug target with versatility for the treatment of the many diseases driven by uncontrolled oxidant production, inflammation and immunologic responsivity [16]. Current clinical applications include autoimmune disease, specifically relapsing remitting multiple sclerosis and psoriasis. There are many other disease indications under clinical and pre-clinical investigation [16]. Our group is currently investigating the fumarate drug, DMF, for therapeutic application in DMD [59], and we are interested in comparing the risk–benefit profile of exogenous (via DMF) versus endogenous (via ASA) fumarate-induced Nrf2 targeting. For example, following 2 weeks oral treatment of DMF (100 mg/kg/day) and ASA (325 mg/kg/day), Nrf2 signaling was induced in DMF [60] but not ASA murine skeletal muscle (C57BL/10 wild-type (WT) and *mdx*; unpublished results). Whereas, 8 weeks treatment via the oral route with a comparable dose did induce Nrf2 expression in *mdx* and WT skeletal muscle [14]. It may be that ASA has less bioavailability than DMF, via the oral route at least. Irrespective, our data suggest that ASA controls a lower grade, and potentially more sustained, Nrf2 activation that may be as effective at attenuating disease with fewer side effects. The appropriate degree of Nrf2 activation required to sustain disease modifying benefits is currently unclear outside of the scope of its clinical use. 

## 4. Medicinal Applications for ASA

Despite the role of the PNC as a critical checkpoint controlling the immuno-metabolic nexus, aside from the ASA trial performed by Bonsett’s team in the context of DMD, ASA has never been investigated for alternative applications. An extensive discussion of ASA’s therapeutic value in DMD, as well as its potential value in treating other diseases, is included here.

### 4.1. DMD

DMD is the only disease for which ASA has been pre-clinically [15] and clinically [1] evaluated. Although not technically an inborn error of metabolism, DMD is characterized by extensive mechanical [7] and mitochondrial [61] perturbations that compromise cellular energy homeostasis and instigate metabolic stress signaling (reviewed in [7,8]). These appear to involve, if not provoke, ROS production, oxidative stress, pathological inflammation and chronic muscle wasting. For example, mitochondrial dysfunction is evident in dystrophin-deficient myoblasts before dystrophin expression normally begins [62] and the transplantation of healthy mitochondria (via muscle stem cell transfers) can significantly improve the phenotype of *mdx* mice [63]. As a result of these metabolic dysfunctions, purine levels (ATP, ADP), and the complementary phosphocreatine pool, are markedly reduced in human DMD and *mdx* skeletal muscles, as is the gene expression of key proteins involved in purine recovery (e.g., hypoxanthine-guanine phosphoribosyltrasferase (HGPRT)). These data indicate significant challenges to purine homeostasis. 

Although there is no direct evidence that ASA levels are altered in DMD, a transcriptomics study of muscles from patients revealed for the first time that *ADSSL1*, which encodes the highly expressed striated muscle specific isoform of ADSS, is significantly downregulated [64]. This infers a reduced capacity for endogenous ASA synthesis that may reduce PNC-mediated purine salvage during metabolic stress leading to persistent purine degradation and intense ROS production by XOR [46]. This scenario would contribute significantly to the oxidative stress, inflammation and chronic hyperactivity of the immune system that drive muscle necrosis, adiposis, fibrosis and loss of function in DMD [7]. The mechanism(s) of PNC downregulation are currently unclear. *ADSSL1* gene expression is reportedly upregulated in cardiac muscle in response to surgically induced cardiomyopathy and angiotensin II, possibly via calcineurin-NFAT signaling [65]. In spinal muscular atrophy type 3 (SMA III) skeletal muscle, *IDH2* and *ADSSL1* are the only under-expressed genes [66] and are associated with a mosaic atrophy and hypertrophy phenotype not too dissimilar from that observed in DMD, where intact muscle fibers hypertrophy to compensate for the loss of strength incurred by atrophying myofibers. Muscle wasting is slowly progressive and ultimately fatal in ADSSL1 deficient myopathy too. These data suggest that *ADSSL1*/ADSSL1 upregulation corresponds with muscle hypertrophy, while downregulation corresponds with muscle atrophy and wasting. *ADSSL1* transcription appears fundamentally linked to the metabolic program associated with biomass accretion over nutrient catabolism, given its role in purine biosynthesis.

There appears to be a distinct mechanism for ASA reprogramming of fat metabolism, at least in the context of DMD. Bonsett’s early work demonstrated that the trace addition of ASA to cultured human DMD muscle explants could correct the mitochondrial TCA cycle defect thought to be mediated by IDH [2,4]. Citrate flux from the TCA cycle is directed into fatty acid synthesis—plausibly, a defective IDH (i.e., due to allosteric inhibition, gene mutation, post-translational modification or altered protein expression), which catalyzes isocitrate > α-ketoglutarate conversion in both the mitochondria and cytosol, would cause citrate accumulation and promote muscle fat synthesis. Lipid droplets could be seen visibly leaching from DMD muscle explants in Bonsett’s culture studies [3]. Modern understanding of the factors that control lipid metabolism and transport within the muscle suggest a more complicated, although congruent, mechanism. For example, genetically dysregulated iron sulfur (Fe-S) cluster formation was shown in mammalian HEK cells to obliterate purine nucleotide biosynthesis—including ASA, IMP, AMP and ADP levels—while driving lipid synthesis and droplet formation [67]. The mechanism was identified as the inhibition of aconitase [67], which not only catalyzes citrate>isocitrate within the mitochondrial TCA cycle, but moonlights as an iron regulatory protein within the cytosol to induce the release of ferritin stores for redox reactions. Skeletal muscle iron stores are increased in at least two murine models of DMD [68], indicating the failure of this mechanism, and iron deprivation is ameliorative [69]. Although it is unclear where the primary metabolic defect lies and how it relates to dystrophin-deficiency, ASA replenishment both in vitro [3] and in vivo [3] can markedly attenuate lipid droplet secretion and muscle liposis, respectively. In fact, over Bonsett’s 10-year clinical trial of ASA completed by one DMD and two BMD patients, reduced muscle fat infiltration and maintenance of function were the most significant benefits of treatment [1]. Although *mdx* mice do not recapitulate muscle liposis as significantly as humans, our pre-clinical work showed moderate-term oral ASA treatment (325 mg/kg/day) also reduced neutral lipid droplets [15]. It is probable that ASA protects against lipid droplet formation via ADSL-mediated fumarate production. As a substrate for FH, fumarate metabolism can overturn aconitase inhibition, leading to a reinstated citrate flux through the TCA cycle and away from lipid synthesis [37]. Furthermore, the aspartate generated in the process stimulates ADSS to re-instate PNC function, purine salvage/biosynthesis and PNC–mitochondrial coupling.

**Figure 4:**
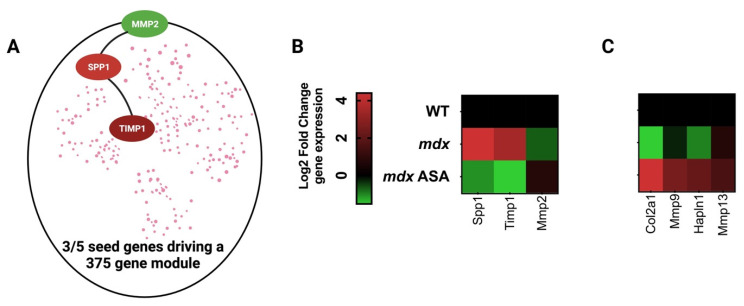
Adenylosuccinic acid (ASA) modulates the Duchenne muscular dystrophy (DMD) disease program. (**A**) Simplified schematic of the DMD disease program generated by Lombardo et al. using a network medicines approach, which is driven by dysregulated expression of matrix metalloproteinase 2 (*MMP2*), secreted phosphoprotein 1 (*SPP1*/osteopontin) and tissue inhibitor of matrix metalloproteinase 1 (*TIMP1*) [70]. (**B**) Heatmap of DMD seed gene expression in *mdx* gastrocnemius muscle. Juvenile (14-day-old) wild-type (WT) and *mdx* mice were treated with either a methyl cellulose (MC) vehicle (*mdx*), or 325 mg/kg/day ASA suspended in MC via oral gavage for 2 weeks. Quadriceps muscles were harvested under non-recovery anesthesia and extracellular matrix gene signature was profiled using qPCR array (for methodology see Supplemental information). (**C**) Heatmap of the ECM genes most modulated ASA. Data shown are log2 fold change expression from WT (for *mdx*) or *mdx* (for *mdx* ASA).

The DMD disease program constitutes thousands of dysregulated genes coordinated by five seed/hub genes, which are mostly involved in ECM pathology [70]. Fibrotic scarring of the ECM is driven by inflammation and oxidative stress, leaving muscles susceptible to hypoxia and nutrient deprivation as myofibers and their capillaries become disconnected. The ECM coordinates the immune response relative to muscle inflammatory signaling, as well as muscle regeneration, via the control of the muscle stem (satellite) cell replication and differentiation [71]. It is particularly sensitive to ROS signaling across the sarcolemma and capillaries (e.g., via XOR) as well as those produced by neutrophils and macrophages [72]. In the first instance, ECM collagen synthesis increases, and matrix remodeling is initiated to facilitate immune cell infiltration, necrotic tissue digestion and muscle repair. However, with chronic immunological activation, the matrix transitions and pathological scar tissue is formed [72]. The dysregulated seed genes driving this transition in DMD include *SPP1*, *TIMP1* and *MMP 2* [70] (Figure 4A). Our data show that ASA can reverse-modulate the expression of all three (Figure 4B), although its biggest impact is on the induction of ECM genes associated with muscle regeneration and hypertrophy (Figure 4C). There are multiple mechanisms that may individually or collectively contribute to this disease-modifying function: (1) by increasing purine salvage, ROS production by XOR is limited, reducing the impact of rampant oxidative stress on the ECM; (2) HCAR2 agonism and Nrf2 activation suppress inflammatory signaling and muscle cytokine production, reducing the impact of rampant immune cell activity on the ECM; and (3) FH activity suppresses ECM collagen synthesis. With respect to point 3, the loss of function mutations in FH are associated with elevated collagen synthesis and tissue fibrosis [37].

Since Bonsett’s ASA trial, therapeutic development for DMD has progressed significantly, and many patients are now being actively treated with exon skipping antisense oligonucleotides and recombinant adeno-associated viral (AAV) vector dystrophin mini/micro-gene restoration therapies in ongoing trials [73,74]. However, despite optimization, dystrophin expression remains relatively low due to the poor bioavailability of oligonucleotides and intrinsic limits of AAV-mediated transduction [75]. Furthermore, micro/mini-gene therapies will never reinstate full-length dystrophin expression leaving patients with a Becker phenotype at best [75]. The low dystrophin levels achieved by these therapies highlight that complementary and synergistic methods are critical to develop. ASA could be particularly useful in this regard.

### 4.2. ADSSL1 Myopathy and Disorders of Purine Synthesis

Of the diseases that could benefit most from ASA therapy, ADSSL1-deficient myopathy is the most likely since endogenous ASA synthesis is severely impaired. ADSSL1 myopathy is an autosomal recessive inborn error of metabolism due to mutations of the *ADSSL1* gene that lead to the loss of function of muscle-specific ADSS [12]. There are <100 reported cases of ADSSL1 myopathy worldwide—predominantly in the Asian population [9,76,77,78,79]—although newly diagnosed (unreported) cases may see the actual number at ~200. As such, ADSSL1 myopathy is classified as an ultra-rare disease and, as with most orphan diseases, there is currently no available therapy to slow progression. At least eight biallelic pathological variants have been identified as causing myopathy, and of these, NM_152328:c.781G>A (p.D261N) and c.919delA (p.I307fs) are the most common, resulting in reduced enzyme function. Every patient with adolescent onset “typical” ADSSL1 myopathy has at least one of four known missense mutations, and a second missense or predicted null mutation. Only one patient with biallelic null mutation has been reported [80], and they presented with fetal akinesia and contractures, suggesting that the complete loss of muscle ADSS leads to a particularly severe phenotype.

Like many metabolic and mitochondrial diseases that manifest in the muscular system, disease onset, severity and progression is variable between patients [78]. However, typical presentation occurs in adolescence during rapid growth when structural stress (stretch across growing bones) and demands on protein synthesis and cellular energy (for growth) are greatest. Clinical symptoms include hypotonia, atrophy and slowly progressive weakness. Although early studies emphasized distal involvement [79], a subsequent larger series documented equal degrees of proximal and distal weakness in more than 50% of patients, and proximal weakness greater than distal in another 14%, making this a generalized disease of all skeletal muscles [78]. By the fourth decade, there is extensive proximal (e.g., quadriceps) muscle involvement. Consistent with the fact that ADSSL1 is expressed at high levels predominantly in skeletal and cardiac muscles, the diaphragm and heart are also affected leading to respiratory insufficiency and cardiomyopathy [78]. Muscle histopathology is characterized by nemaline rods and significant lipidosis [76]—both features have been linked to metabolic stress in vitro although the mechanisms remain unclear [77]. Centronucleated fibers of variable size, fiber splitting, rimmed vacuoles and focal fibrosis are also observed [76,77,81]. Severe late-onset nemaline myopathy (SLONM) is a non-genetic form of rod myopathy associated with autoimmunity [82], and it is likely that inflammation and hyper-immunity could be involved in ADSSL1 myopathy given purine deregulation is linked to other autoimmune diseases [83]. Mild elevations in CK [77,81] indicate muscle wasting is, at least in part, due to necrosis or to a propensity for mechanical damage/membrane disruption. A recent case study reported swollen mitochondria in the muscle biopsy of a 15-year-old Korean female ADSSL1 patient [81], a symptom of calcium dysregulation secondary to metabolic and oxidative stress [8].

ASA replenishment could be medicinally valuable for ADSSL1 myopathy as it is the primary reaction product generated by ADSSL. Theoretically, it could re-establish PNC-mediated recovery of IMP to (1) ensure purine and energy homeostasis; and (2) limit ROS production and inflammation instigated by XOR-mediated purine degradation. Furthermore, ASA could promote the functional recoupling of the PNC and mitochondria and facilitate end-stage purine biosynthesis. All of these functions are essential for biomass accretion over degradation, and the dysregulation of them likely drives the myopathy. ASA may also be clinically useful for the five additional non-lethal ultrarare disorders linked to the loss or gain of function mutations of purine biosynthesis/salvage enzymes (summarized in Table 1; for an excellent review of these disorders see [12]). These disorders share clinical symptoms more synonymous with primary mitochondrial disorders than myopathies per se, suggesting that at least part of their pathological complexity is due to the loss of mitochondrial coupling.

### 4.3. Diabetes

Increasingly, diabetes and obesity are linked to a dysregulated purine metabolism. SAICAR (the substrate of ADSL function within the de novo purine biosynthesis pathway) and xanthine are elevated in the plasma of obese and Type 2 diabetes patients [84]. However, urinary profiles differ between these cohorts. Whereas uracil is elevated in obese patients, SAICAR and AICAR are elevated in Type 2 diabetes [84]. These findings suggest that purine degradation is elevated in obesity and perhaps pathologically so in Type 2 diabetes. In skeletal and cardiac muscle, and in the liver of diabetic rats, an increased catabolism of AMP is evident resulting in decreased high-energy purines (ATP, ADP and AMP) and increased low-energy purines (IMP) [85]. ADSS and ADSL activity are markedly increased [85], indicating PNC function may become overwhelmed.

In the end stage of Type 2 diabetes development, pancreatic insulin secretion, and the organism’s capacity to transport glucose into storage tissues such as skeletal muscle and the liver, is lost. An increased ASA and reduced IMP is correlated with this loss of function [84], suggesting an inhibition of ADSS (and potentially ADSL) is involved. Thus, there are distinct, tissue-specific changes to purine metabolism within the pancreas compared to glucose disposal tissues [84]. Intriguingly, the exogenous application of ASA to diabetic β-islet cultures could overturn this dysfunction of glucose-stimulated insulin secretion [25], suggesting that: (1) the inhibition of ADSS/ADSL is allosteric; and (2) that ASA is an important regulator of insulin secretion and glucose disposal. The upregulation of ADSS function in diabetic glucose storage tissues (muscle, liver) [84] indicates that ASA might also be crucial for the control of glucose uptake and flux into the purine biosynthesis pathway. Diabetic muscles display varied degrees of myopathy, including weakness, atrophy and liposis, consistent with other disorders of purine nucleotide metabolism. While diabetic muscles appear not to be PNC-deficient per se, their over-reliance on purine metabolism to meet basic energy needs may leave them predisposed to pathology as per purine metabolism disorders. ASA could thus be a valuable therapeutic to address various aspects of Type 2 diabetes on multiple levels.

### 4.4. Other Applications

Since all cellular metabolic systems are ultimately linked, and the pharmacological manipulation of just one is likely to have flow-on effects across the entire cell, ASA—depending on its precise mechanism of action—may be useful to treat a variety of other diseases. The pathogenic variants of the *SELENON* gene, encoding selenoprotein N, have been shown to cause a muscle disease characterized by muscle weakness, spinal rigidity and respiratory insufficiency [86]. Selenoprotein N is an endo/sarcoplasmic reticulum (ER/SR) membrane protein, enriched in contact areas between the ER and mitochondria [87]. A lack of selenoprotein N leads to impaired ER/mitochondria contacts and decreased ATP levels. Cellular and mouse models knock-out for *Selenon* gene present increased susceptibility to ER stress, resulting in a lower metabolic rate [87]. ASA treatment may be beneficial in *SELENON*-related myopathy, as well as in other metabolic myopathies, by boosting mitochondria activity and recycling IMP. 

Neurodegenerative diseases, such as Parkinson’s and Friedrich’s Ataxia, have been historically associated with oxidative stress-mediated changes to the brain. However, more recently, these disorders have been etiologically linked to Fe-S cluster dysfunction [88]. Although it is unclear whether ASA specifically modulates iron homeostasis or Fe-S cluster formation, it can beneficially modulate many of the downstream implications of Fe-S cluster defects, e.g., lipid droplet formation [67], and could, therefore, be useful in treating the consequences of that etiology. The mechanisms seem likely related to Nrf2 induction, since Nrf2 controls both iron and redox homeostasis [89], and the Nrf2 activator, DMF, can modulate an extensive lipidomic profile [90]. If ASA imparts its biological benefits solely through the fumarate-dependent activation of Nrf2, it could be used as an alternative pharmaceutical for diseases clinically treated with fumarate ester therapy, e.g., multiple sclerosis and psoriasis. In this context, ASA might exert better and safer control over Nrf2ome induction through prompting endogenous fumarate synthesis that is proportionate to both aspartate production and FH expression. Indeed, the over-succination of proteins by chronically high cellular fumarate levels is associated with tumorigenesis [24]. Although the mechanisms are currently unclear, *ADSSL1* expression [65] and purine nucleotide metabolism [91] are also altered in cardiomyopathy and heart failure—ASA treatment could be therapeutically beneficial in these conditions.

## 5. Methods

Mature messenger RNA was isolated from quadriceps homogenates using the RNeasy1 mini kit (Qiagen, Hilden, Germany) according to the manufacturer’s instructions. Cell lysates were transferred onto RNeasy mini-spin columns and DNA was removed using DNase digestion/treatment using RNase-Free DNase Set (Qiagen, Hilden, Germany.) The RNA Integrity Number (RIN) of all samples was quantified using an Agilent 2100 Bioanalyser and Agilent RNA 6000 nano kit (Agilent Technologies, Santa Clara, CA, USA). RIN values above 7.5 were used as the inclusion criterion for subsequent gene expression analysis. The concentration of RNA samples was measured using a Qubit RNA BR Assay (Invitrogen, Waltham, MA, USA) in triplicate. Aliquots of each RNA sample were reverse-transcribed to make complementary DNA (cDNA) using an RT2 first strand kit (Qiagen, Hilden, Germany) according to the manufacturer’s instructions. A quantitative real-time polymerase chain reaction (qRT-PCR) was performed using the Qiagen Mouse Inflammatory Response and Autoimmunity (PAMM-077Z) and Mouse Extracellular Matrix and Adhesion Molecules (PAMM-013Z) RT2 Profiler PCR arrays (Qiagen, Hilden, Germany) to evaluate the relative gene/mRNA expression in WT compared to mdx and in treated compared to untreated muscles. CT values were normalised based on a selection of reference genes (ACTB, B2M, GAPDH, GUB, HSP90AB1) and fold changes/regulation of gene expression were calculated using the 2^(-DDCT) formula (GeneGlobe, QIAGEN). Differential expression (up and down regulation) genes were identified using the criteria of a >1.5-fold increase/decrease in gene expression, *p* < 0.05 from reference group. Heatmaps were created using Log2-transformed Z scores using Prism 9.

## 6. Conclusions

Although an orphan drug, the novel insights gleaned in the past decade concerning the dynamic crosstalk between the metabolic and immune systems and integrative stress pathways that promote cell survival, especially the Nrf2ome, beg a new assessment of the potential clinical utility of ASA. The dysregulation of purine metabolism and biosynthesis has been linked to many diseases across multiple organ systems and could be pharmacologically exploited to manage downstream events, especially to manage oxidative stress, inflammation and chronic immune system activation. While ASA’s synthetic and degradative enzymes are most active in skeletal and cardiac muscle, suggesting diseases of these systems stand to benefit most from ASA therapy, there may be a broader application given ASA’s Nrf2-activating and insulin-secreting functions. There may well be a business case for the large-scale production and testing of ASA in translational medicine. Future clinical trials e.g., for ADSSL1 myopathy, will benefit from the safety and dose–response data already collected in Bonsett’s DMD/BMD trials. However, the economical large-scale sourcing of ASA could remain an obstacle to patient access if future clinical trials are successful. Further studies are required to decipher pharmaco-kinetic and -dynamic information and to ultimately determine the delivery-route-specific dose required to replenish deficient ASA within target tissues.

## Figures and Tables

**Table 1:** Rationale for therapeutic treatment of purine disorders with ASA. Key: IMP = inosine monophosphate; PNC = purine nucleotide cycle; ASA = adenylosuccinic acid; UA = uric acid; HPRT = hypoxanthine-guanine phophoribosyltransferase; AMP = adenosine monophosphate; ADSL = adenylosuccinate lyase; SAICAR = phosphoribosylaminoimidazolesuccinocarboxamide; AICAR = 5-aminoimidazole-4-carboxamide ribonucleotide; ADSSL1 = adenylosuccinate synthetase like-1; ADSS = adenylosuccinate synthetase; XMP = xanthosine monophosphate.

Disease	Potential ASA Benefit	Proposed Mechanism
Phosphoribosylpyrophosphate synthetase overactivity	Possible	Excessive IMP via de novo biosynthesis which overwhelms PNC function resulting in degradation and inflammation—ASA supports IMP conversion to fumarate and AMP to reduce degradation to UA and inflammation.
Phosphoribosylpyrophosphate synthetase deficiency	Possible	Insufficient IMP produced through biosynthesis and reduced HPRT-mediated salvage activity—ASA supports IMP salvage conversion to fumarate and AMP via the PNC.
Bifunctional enzyme phosphoribosylaminoimidazole succinocarboxamide synthetase deficiency	N/A	Lethal immediately after birth.
Adenylosuccinate lyase (ADSL) deficiency	Possible	ADSL converts ASA to AMP and fumarate. Depending on the levels of functional enzyme present (dictating the severity of disease), increasing ASA levels may amplify endogenous ADSL catalysis rate, although ASA levels are usually elevated in ADSL deficiency.
AICAR transformylase/IMP cyclohydrolase deficiency	Possible	Insufficient IMP produced through biosynthesis and build-up of SAICAR and AICAR, indicating ADSL function is compromised—ASA supports IMP salvage, ADSL enzyme activity and conversion to fumarate and AMP via the PNC.
Adenylosuccinate synthase muscular isoform deficiency (ADSSL1 myopathy)	Likely	ASA synthesis by ADSSL1 is reduced relative to endogenous ADSS expression reducing substrate availability for ADSL. Exogenous ASA replenishment would restore ADSL, and PNC, function.
Inosine triphosphatase deficiency	Unlikely	Disease is associated with impaired removal of non-canonical (deoxy-) nucleotide triphosphates (IMP and XMP) rather than IMP metabolism per se.

## Data Availability

Not applicable.
